# The Impact of Hoarding Disorder on Family Members, Especially the Significant Other

**DOI:** 10.7759/cureus.45871

**Published:** 2023-09-24

**Authors:** Anureet K Sekhon, Luba Leontieva

**Affiliations:** 1 Psychiatry, State University of New York Upstate Medical University, Syracuse, USA

**Keywords:** family, compulsive hoarding, spouse, clutter, hoarding disorder

## Abstract

Hoarding disorder, also known as compulsive hoarding, comes under the umbrella term of obsessive-compulsive disorder and related disorders. The constant building of clutter in the house of hoarders makes it impossible for family members to live a healthy life. It can have appalling effects on their mental health and can lead to severe depression and suicidal ideation. The shame and humiliation attached to hoarding does not allow the hoarders to seek help, causing them distress and hence continuing the vicious cycle of hoarding. By presenting the case of a patient with a spouse with a hoarding disorder, we want to bring to light the severity of the impact hoarding disorder can have on the partner and the grave need to spread awareness about it so that the patient or the family members can seek timely help.

## Introduction

Compulsive hoarding is a rare disorder seen in 2%-5% of the adult population [1] and was recently added to the Diagnostic and Statistical Manual of Mental Disorders, Fifth Edition (DSM-5). It is likely under-reported as people who are hoarding often do not come to see a mental health professional as they lack awareness of the problem (anosognosia) [2]. Patients with hoarding disorder have persistent difficulty discarding their possessions regardless of their value to them. The act of discarding items gives rise to feelings of overwhelm and anxiety, and the inability to do so is known to be associated with a significant amount of shame and distress to the hoarders. The persistent collection of items leads to the building of huge amounts of clutter in their houses and miserable living conditions [3].

A large number of patients may lack awareness of their problem [2], which can cause their loved ones to resent them and can lead to growing frustration among family members. On the contrary, some individuals with the disorder may have insight into their disease but may be unable to help themselves against the compulsions, causing severe debilitation. Patients may also exhibit behaviors such as difficulty making simple decisions, the need to do certain chores perfectly, delaying task completion, and disorganization [4]. These factors, along with the enormous amount of guilt and humiliation associated with compulsive cluttering, can have a harmful influence on the mental well-being of hoarders [5]. Family members often fail to convince the hoarders to see the intensity of the problem and seek help. This can cause straining of intrafamilial relationships [1,6], especially between spouses living in the same house, leading to an increased number of broken families and an escalated rate of failed marriages [7]. It keeps progressing with time until brought to attention and becomes difficult to treat as the severity of the disease increases [8]. Overall, hoarding disorder can cause remarkable deterioration in the social, physical, and mental functioning of the individual [5,9]. However, very scant literature is available in context to the impact of hoarding on the spouse and the ways to better address the issue.

The goal of this article is to shed light on the drastic impact of hoarding on family members (especially the significant other) and the dire need to come up with better ways to manage the issues faced by the patients and their loved ones by presenting the case of the spouse of a hoarder, an elderly male who was diagnosed with severe depression and was admitted after a very serious suicide attempt.

This study is a descriptive case report that aims to shed light on this rare condition that drastically affects the family members and requires social interventions in the community, which are non-existent based on our attempts. This study was conducted at an acute inpatient psychiatric floor. The patient started on electroconvulsive therapy (ECT) after one month of antidepressant, antipsychotic, and benzodiazepine treatments. As of the time of this report, he received eight sessions of ECT with some improvement. The target is 12 sessions. The follow-up of his wife getting the house decluttered remained poor. No agencies can check on the house and help her.

## Case presentation

We present a case of a 76-year-old retired professional Caucasian male with a recent history of depression and one recent suicide attempt by cutting his wrist following hospitalization admitted to the emergency department (ED) with a very serious second suicide attempt by stabbing himself in the abdomen with a knife. His suicide attempt was triggered by the fact that his wife was a hoarder.

He said his privately owned house is cluttered with stuff that his wife keeps buying. Conditions at home were described as unlivable by the patient due to hoarded items making piles up to counter height or higher. He felt his home was not a safe place for both of them and reported having difficulty getting around in the house. Entrances were blocked, the shower was inaccessible, and the workspace was filled with boxes and stuff cluttered in all the available living space in the house. The patient was unsure if the food he was consuming was healthy and edible because of abundant food rotting in the house. There was little room to move around, and garbage was present throughout the house. The patient was worried about the amount of money being spent on useless items and reported they might be in debt. This led to a difficult psychosocial situation for the patient.

He was concerned about his wife’s safety and wanted her to get help. He said he tried to convince her to see the problem, but she lacked insight into it. She would make excuses and sometimes even agree to make a change, but she never could keep her word because it was stressful for her to give up her possessions. He said he tried to threaten to leave her, but she knew better. He said the hoarding started 20 years from now but was not extreme at the time. It progressed over time, and over the past couple of years, it has escalated, leading to a living crisis at home. All these situations led him to feel so helpless and hopeless that he decided to take his life, thinking things were never going to get better.

According to him, his wife was very secretive about hoarding and would resist calling for outside help even in urgent situations. He described an incident when she had a fall because of the clutter and would not let her husband call for help because she did not want people to see the house in this condition until it was extremely unavoidable.

After retirement eight years ago, the patient stayed mostly at home, which made it more difficult for him to adjust to the settings and added to his feeling of helplessness, leading him to attempt to end his life.

Family history

There was no history of mental illness or suicide in the family.

Past psychiatric history

The patient has no history of psychiatric illness. He has never seen a psychiatrist or been admitted to any psychiatric units before. The patient’s first and most recent encounter with the psychiatry unit was one month before presentation when he cut his wrist in an attempt to kill himself due to the same stressors at home related to his wife’s hoarding.

Past medical history

The patient has the following medical comorbidities (along with related medication): hypertension (on amlodipine), benign prostatic hyperplasia (on tamsulosin), chronic obstructive pulmonary disease (on fluticasone-salmeterol and albuterol inhalers), gastroesophageal reflux disease (on Protonix), hypercholesterolemia (on atorvastatin), chronic constipation (on various bowel medications), and mixed conductive and sensory hearing loss.

Personal and social history

The patient is a retiree and has been married for 52 years to his current wife. They exhibit characteristics of codependency on one another. The patient had no strength to go through the piles of accumulated items and hence was dependent on his wife to go around the house to get food. The wife on the other hand was dependent financially on the patient.

The couple do not have children and live together in a house. The patient has a sister who has not visited the couple in a long time and lives across the country. The patient does not have friends who visit them as his wife does not allow people in the house.

Interventions in the psychiatry unit

The patient was transferred to the psychiatry unit from surgery after medical stabilization. The patient had been on mirtazapine 30 mg after the last suicide attempt one month before the current presentation. The patient was continued on mirtazapine 30 mg initially, which was tapered off for non-effectiveness, meanwhile adding escitalopram, which was titrated to 20 mg daily (maximum dose for elderly). The patient was evaluated by a neuropsychologist.

The following is the list of tests administered for neuropsychological testing: Beery Visual-Motor Integration (VMI) test, Boston Naming Test (BNT), Brief Visuospatial Memory Test-Revised (BVMT-R), Clock Drawing Test, Delis-Kaplan Executive Function System (D-KEFS) (Verbal Fluency and Color Word), Hopkins Verbal Learning Test-Revised (HVLT-R), Neuropsychological Assessment Battery (NAB) (Orientation and Visual Discrimination), Test of Premorbid Functioning (TOPF), Trail Making Test (Part A and B), Wechsler Adult Intelligence Scale-Fourth Edition (WAIS-IV) (Digit Span, Similarities, and Matrix Reasoning), and Wechsler Memory Scale-Fourth Edition (WMS-IV) (Logical Memory).

The neuropsychologist did not confirm dementia based on comprehensive testing, but executive dysfunction was diagnosed due to his severe depression. The patient was initiated on olanzapine to augment the escitalopram and to target his mood-congruent psychotic symptoms such as reports of hearing voices telling him he is bad. He improved initially with the addition of olanzapine 2.5 to 5 mg daily. However, after a week of treatment, he started to display catatonic behaviors: mutism, posturing, and standing in one position. Due to these symptoms, olanzapine was stopped, and lorazepam was initiated on 0.25 mg dose, which was increased to 0.5 mg nightly. The patient has very slow minimal improvement with this regimen. Since he failed trials of mirtazapine and later escitalopram augmented with olanzapine and lorazepam separately, he was declared treatment-resistant for depression. Therefore, he was consulted for electroconvulsive therapy (ECT) and was found to be a suitable candidate for it due to his treatment-resistant depressive disorder and catatonic symptoms. After eight sessions, there is an improvement in his depression. At the time of this report writing, he was started on ECT and had received eight sessions with improvement in his depression.

While in the inpatient unit, multiple efforts were made to help the wife declutter and clean the house. Adult protective services was contacted, and they refused to take the case, stating that the patient was safe in the hospital. Extra cleaning help by community agencies was offered but was failed to be utilized by the wife. Multiple family meetings were held with the wife present, during which she promised to clean and declutter but never did.

The code enforcement agency was contacted, and the response was that they could not help because the patient and his wife own the home. Since no one has seen the state of the home, they could only send a letter telling the patient and his wife they were notified of a complaint and encourage them to fix it themselves.

## Discussion

This case sheds light on the seriousness of hoarding disorder and the severity of the impact it can have on family members, especially the significant other. Hoarding behavior is known to start early in life, but the intensity may not be significant for diagnosis at a younger age. It progresses over time and becomes evident later in life, which is why most of the patients present in old age when the disease becomes severe [4] and difficult to treat [8].

Hoarding disorder can have a wide array of effects on the health and well-being of family members. It causes straining of relationships among family members [10]. Hoarding can lead to a wide range of serious problems. Inaccessibility of daily necessities such as showers, toilets, kitchens, and working stations affects the social and physical health of all the members living in the house [6]. Hoarding can cause serious physical harm such as tripping over things, which can cause injuries ranging from minor scratches and bruises to serious fractures and deep hemorrhagic wounds. Collection of papers, cardboard, and other inflammable objects can lead to potential fire hazards. Hoarders may resist receiving emergency care after getting hurt or due to other health issues, because of excessive embarrassment involved with compulsive cluttering. Various health problems can occur due to mold and pests growing on the hoarded perishable items. Ignorance and hence persistence of these conditions for prolonged periods can cause damage to property [11].

There is often an excessive amount of shame associated with hoarding, which can prevent people from having guests at home, leading to social isolation, which in the long run can lead to depressive feelings [6]. Often, hoarders are left alone to deal with the problems, aggravating the feeling of isolation and defeat [1]. Due to associated shame and guilt, hoarders may not seek help, and they tend to resist family members’ persuasion to seek treatment. The family members may develop feelings of growing frustration and resentment toward the hoarders, making them vulnerable to psychiatric illnesses such as depression and anxiety. This leads to strained interpersonal relationships, family discord [6], decreased feelings of happiness, decreased quality of life, increased chances of divorce, and broken marriages [7].

Conventional treatment options such as cognitive behavioral therapy (CBT) and pharmacotherapy including selective serotonin reuptake inhibitors (SSRIs) have proved to be less effective in treating hoarding disorder. This difficulty associated with treatment could be related to the presence of masked underlying comorbidities [8] that render the conventional treatment options less effective. The neurocognitive deficit witnessed in many patients with hoarding disorder [12] is known to limit the effectiveness of standard CBT [13]. The goal is to address underlying psychosocial stressors and familial conflicts for better outcomes [8].

There can be a significant economic burden linked to hoarding. Patients are more prone to losing jobs and may fail to file income tax at times [3]. Our patient complained of being in debt, which added to his suffering.

Altogether, hoarding disorder can impair the social, physical, and emotional functioning of not only the hoarders but also their relatives. Our goal is to spread awareness about the severity of the impact of hoarding so that family members can recognize patterns early in the disease and seek timely help. The agencies and rules already in place responsible for taking care of this situation may prove to be insufficient as in our case, which can increase desperation among family members and force them to take drastic steps.

Our patient presented with a self-inflicted stab wound in his abdomen in an attempt to kill himself to get rid of the hoarding situation at home, which reflects the seriousness of the condition. He had been barred from using the shower, toilet, workspace, and even kitchen due to clutter for a long time. He persistently pleaded and later threatened his spouse to address the issue, but the spouse kept on postponing to address it. His inability to pacify his spouse and growing feelings of hopelessness and helplessness rendered him to attempt suicide. If the patient gets better with his depression to the point of being discharged home, such discharge could not be accomplished due to the unavailability of his house and unsafe conditions.

## Conclusions

Hoarding disorder has a detrimental impact on spouses and other family members. The enormity of the impact on a spouse is evident in our case report. This article brings to light the need to establish efficient methods to identify mental health issues in family members of hoarders and to put in place effective management strategies that benefit both parties so that the hoarders can get timely treatment before reaching extremes of the disorder and the suffering of family members leading to the development of mental health disorders in them can be prevented or at least be managed at early stages. Spreading awareness about the availability of these management policies should be considered an area of focus. The message for abstaining from normalizing psychiatric issues in family members needs to be sent across society to help destigmatize seeking help for these issues.
